# High prevalence of endometriosis in women with chronic pelvic pain and confirmed pelvic varicosities: a multiparametric MRI study

**DOI:** 10.1016/j.clinsp.2026.100895

**Published:** 2026-03-03

**Authors:** Mira Zlotnik, Marcos de Lorenzo Messina, Thiago Dieb Ristum Vieira, Vinicius Bertoldi, Andrei Skromov de Albuquerque, Pedro Puech-Leão, Edmund Chada Baracat

**Affiliations:** a Gynecology Discipline, Department of Obstetrics and Gynecology, Hospital das Clínicas, Faculdade de Medicina, Universidade de São Paulo (HCFMUSP), São Paulo, SP, Brazil; b Department of Radiology, Hospital das Clínicas, Faculdade de Medicina, Universidade de São Paulo (HCFMUSP), São Paulo, SP, Brazil; c Department of Vascular Surgery, Hospital das Clínicas, Faculdade de Medicina, Universidade de São Paulo (HCFMUSP), São Paulo, SP, Brazil

**Keywords:** Chronic pelvic pain, Endometriosis, Pelvic varicosities, MRI, Non-Invasive Imaging

## Abstract

**Objective:**

Chronic Pelvic Pain (CPP) presents significant diagnostic challenges, often involving multiple pathologies, including endometriosis and pelvic venous disorders. This study examined the prevalence of endometriosis among women with confirmed pelvic varicosities.

**Methods:**

The authors performed a retrospective analysis of 52 women with CPP and pelvic varicosities who underwent multiparametric magnetic resonance venography between January 2019 and December 2023. All images were reviewed by a single experienced radiologist, blinded to clinical data and vascular findings, to identify radiological signs of endometriosis.

**Results:**

Among the 52 women, 32 exhibited radiological findings consistent with endometriosis, corresponding to a prevalence of 61.5% (95% CI: 47.0%–74.5%).

**Conclusion:**

This study demonstrates a high prevalence of endometriosis among women with pelvic varicosities. Multiparametric MRI proved effective as a non-invasive diagnostic modality capable of identifying both vascular and gynecologic abnormalities in a single session, supporting integrated assessment approaches for women with CPP. Limitations include the retrospective design and the lack of histological confirmation of endometriosis.

## Highlights

• Endometriosis was found in 61.5 % of women with chronic pelvic pain and confirmed pelvic varicosities.
• Multiparametric MRI allows non-invasive, simultaneous detection of vascular and gynecological abnormalities.
• Integrated imaging may improve diagnostic workup in women with chronic pelvic pain.

## Introduction

Chronic Pelvic Pain (CPP) is defined as intermittent or constant pain in the lower abdomen or pelvis lasting at least six months, not occurring exclusively with menstruation or intercourse.^1^ This condition is frequently associated with negative cognitive, behavioral, sexual, and emotional consequences, as well as symptoms of lower urinary tract, sexual, bowel, pelvic floor, myofascial, or gynecological dysfunction.^2^ This multifactorial condition affects 5.7 % to 26.6 % of women globally.^3^ and requires a multidisciplinary approach addressing both physical manifestations and psychological impacts on quality of life.^1^^,^^2^

CPP encompasses a broad differential diagnosis, with endometriosis and Pelvic Venous Disorders (PeVD) among its leading etiologies.^4^^,^^5^ Endometriosis is characterized by ectopic endometrial tissue and typically presents with dysmenorrhea, dyspareunia, dyschezia, and infertility.^5^ PeVD, also referred to as pelvic congestion syndrome, is frequently underdiagnosed and presents with non-cyclical pelvic pain, postcoital dyspareunia, and visible vulvar or lower limb varicosities.^4^

The reported prevalence of endometriosis varies considerably depending on the population studied and diagnostic methods employed. A systematic review by Moradi et al. reported a global prevalence of 18 %, reaching 42 % in women with CPP and 31 % in those with infertility.^6^ Harder et al. identified prevalence rates of 0.76 % in administrative datasets, 6.8 % in clinical studies, and up to 21 % among symptomatic women.^7^ In a cross-sectional study of 373 symptomatic women, Orlov & Jokubkiene diagnosed endometriosis in 25 % using expert-performed transvaginal ultrasound.^8^

**Fig. 1:**
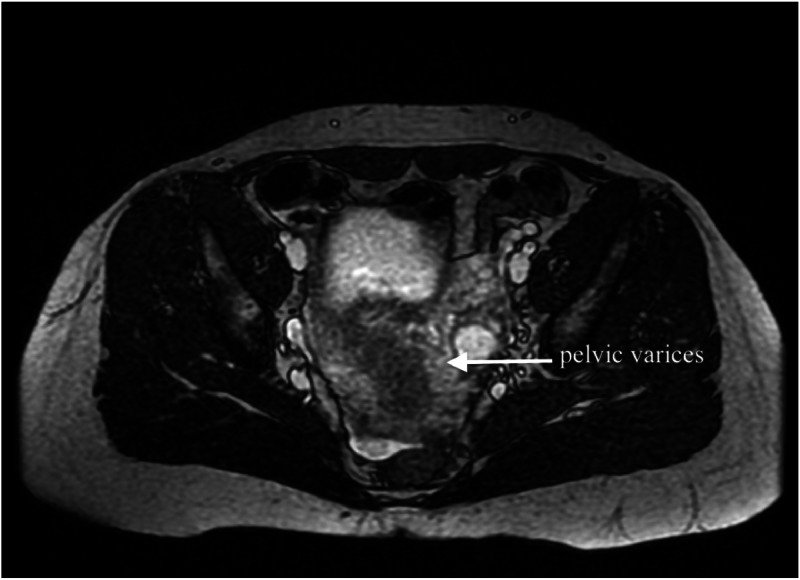
Axial T2-weighted MRI showing dilated parauterine veins (arrow) consistent with pelvic varicosities. These venous dilatations are characteristic findings of pelvic venous insufficiency in patients with chronic pelvic pain.

**Fig. 2:**
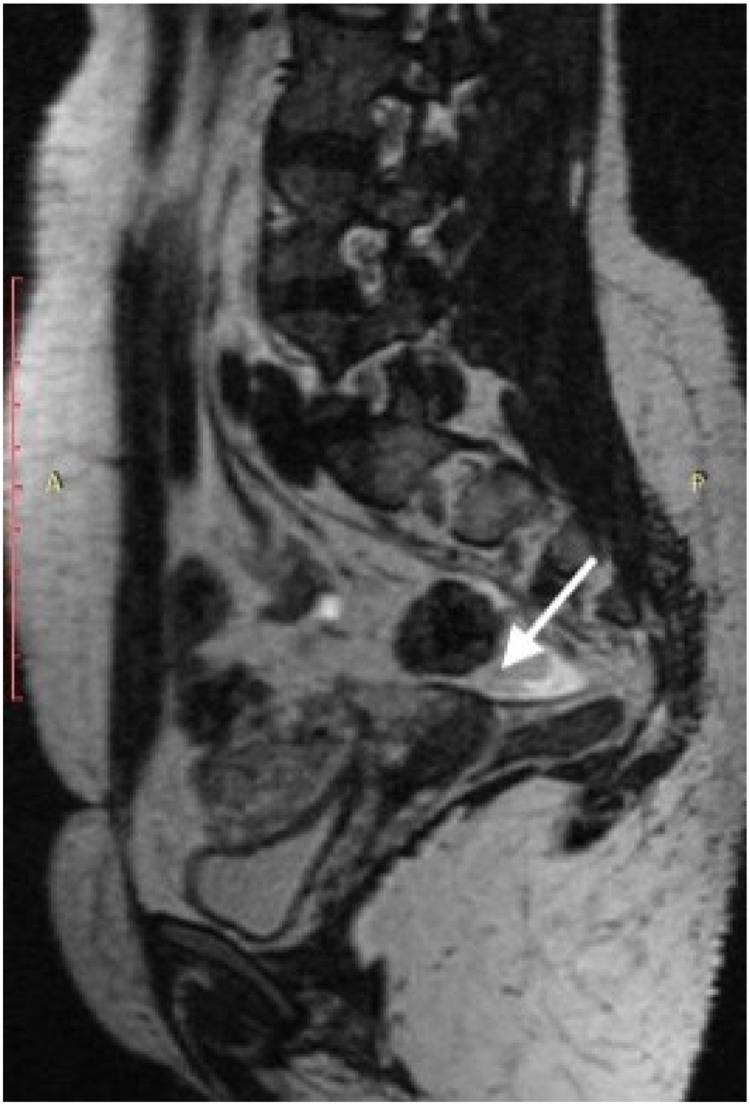
Sagittal T2-weighted pelvic magnetic resonance image showing a low-signal-intensity thickening in the retrocervical region, extending to the left paracervical area and left uterosacral ligament (arrow), consistent with deep infiltrating endometriosis.

Given the limitations of laparoscopy and venography ‒ the traditional gold standards for diagnosing endometriosis and pelvic venous disorders, respectively ‒ non-invasive imaging techniques have gained increasing relevance. Multiparametric Magnetic Resonance Imaging (MRI) offers the advantage of evaluating both vascular and gynecologic structures in a single session and has demonstrated good sensitivity for detecting deep endometriosis and pelvic venous insufficiency.^9^^,^^10^ (Fig. 1, Fig. 2).

This study examined the prevalence of endometriosis in women with confirmed pelvic varicosities, considering the clinical overlap between these two conditions. While previous literature reports endometriosis rates of 21 %–42 % in symptomatic women,.^6^, ^7^, ^8^ the frequency of coexistence with pelvic venous disorders remains unclear. By specifically analyzing women with confirmed pelvic varicosities, this study aimed to determine the proportion of patients who also present with imaging findings of endometriosis.

## Materials and methods

This retrospective observational study included women with chronic pelvic pain and pelvic varicosities who underwent multiparametric magnetic resonance venography (MRV) for evaluation of pelvic venous congestion. Pelvic varicosities were defined by congestion of the uterine venous plexus, characterized by multiple tortuous myometrial veins > 5 mm in diameter.

All MRV scans were reviewed by a single experienced radiologist to identify radiological signs of endometriosis. The reviewing radiologist was blinded to the clinical data and prior diagnoses, as well as to the vascular findings of the MRI and evaluated volumetric coronal T2-weighted sequences with multiplanar reformations, as well as sagittal and axial T1-weighted sequences before and after contrast administration. Patients were included if they presented with chronic pelvic pain (pain lasting ≥6-months) and confirmed pelvic varicosities, had undergone MRV between January 2019 and December 2023, and had complete imaging data available.

Endometriosis was diagnosed radiologically based on characteristic imaging findings following established diagnostic criteria. Specific findings included low T2-signal thickening in typical locations such as the posterior uterine serosa, uterosacral ligaments, rectovaginal septum, paracervical regions, ovarian capsules, and other deep pelvic compartments.

Descriptive analysis was used to determine the prevalence of endometriosis among patients with pelvic varicosities. Continuous variables, including age, number of pregnancies, and symptom intensity scores, were compared between groups using the Mann-Whitney U test. Categorical variables were analyzed in terms of their prevalence using Chi-Square or Fisher's Exact tests. Symptom intensity (chronic pelvic pain, dysmenorrhea, and dyspareunia) was assessed using a visual analog scale (VAS) ranging from 0 to 10. Statistical significance was set at *p* < 0.05. All analyses were performed using STATA 16-SE.

This retrospective observational study was conducted in accordance with the STROBE Statement for observational studies. This study was approved by the Institutional Research Ethics Committee of Hospital das Clínicas, Faculdade de Medicina da Universidade de São Paulo (CAAE: 86,268,625.0.0000.0068), in accordance with the principles outlined in the Declaration of Helsinki and national guidelines for research involving human subjects. The retrospective nature of the study and the use of anonymized imaging and clinical data were considered by the ethics committee.

## Results

A total of 52 women with chronic pelvic pain and pelvic varicosities were included, with a mean age of 34.9-years. Table 1 describes the characteristics of the groups with and without endometriosis. Continuous variables, including age, number of pregnancies, and symptom intensity scores (chronic pelvic pain, dysmenorrhea and dyspareunia assessed by visual analog scale) were compared between groups using the Mann-Whitney *U* test. No statistically significant differences were observed between groups for any of the analyzed variables (*p* > 0.05 for all comparisons), demonstrating the clinical similarity of both conditions. The absence of statistically significant differences may reflect limited power due to the small sample size.

**Table 1:** Demographic and clinical characteristics according to endometriosis presence.

	Without endometriosis (n = 20)	With endometriosis (n = 32)	p-value^a^
	Mean (± SD)	Mean (± SD)	
Age (years)	33.36 (±8.04)	35.65 (±7.59)	0.334
Pregnancies	2.27 (±1.48)	2.06 (±1.36)	0.596
CPP (VAS score)	8.76 (±1.14)	8.54 (±2.04)	0.856
Dyspareunia (VAS score)	8.35 (±2.52)	8.00 (±3.07)	0.99
Dysmenorrhea (VAS score)	7.82 (±2.96)	5.70 (±4.25)	0.121

a Mann-Whitney U test. SD, Standard Deviation; CPP, Chronic Pelvic Pain; VAS, Visual Analog Scale (0–10).

All patients had undergone multiparametric MR venography for clinical evaluation of pelvic venous insufficiency (Fig. 1). Among them, 32 patients exhibited radiological findings consistent with endometriosis, corresponding to a prevalence of 61.5 % (95 % CI: 47.0–74.5 %) (Fig. 2). These findings were assessed by a single experienced radiologist.

Although histological confirmation was not available, imaging interpretation followed established diagnostic criteria for endometriosis in deep pelvic compartments. No adverse events or technical limitations were reported during image acquisition. The ability to simultaneously identify signs of both endometriosis and pelvic venous congestion in a single imaging session demonstrates the feasibility of a multiparametric approach in routine clinical practice.

## Discussion

The present analysis demonstrated that 61.5 % of women with confirmed pelvic varicosities had radiological signs of endometriosis, suggesting frequent coexistence of these conditions in women with chronic pelvic pain.

The prevalence of endometriosis observed in this cohort (61.5 %) exceeds the range reported in the literature for symptomatic women (21 %–42 %).^6^, ^7^, ^8^ Although no formal statistical comparison with historical controls was performed, this finding suggests a high degree of coexistence between endometriosis and pelvic varicosities and warrants further investigation in controlled prospective studies.

Both conditions share certain clinical presentations and anatomical distributions within the pelvis. Previous research has documented inflammatory markers in both endometriosis and pelvic venous disorders. Studies have reported associations between hematological inflammatory markers and endometriosis severity,.^11^ while elevated concentrations of inflammatory mediators such as IL-6 and C-reactive protein have been identified within varicose ovarian veins.^12^ These observations suggest that systemic and localized inflammatory processes may be present in both conditions, though the nature of any relationship remains to be elucidated.

The high co-occurrence observed in this study emphasizes the importance of comprehensive diagnostic approaches that consider multiple overlapping pelvic pathologies rather than focusing on isolated conditions. Multiparametric MRI enabled simultaneous identification of both vascular and gynecologic abnormalities, supporting its role as an integrated assessment tool.

While laparoscopy and venography remain gold standards for definitive diagnosis, they are invasive procedures with limited accessibility. MRI has demonstrated high sensitivity in detecting both deep infiltrating endometriosis (76 %–96 %) and pelvic venous insufficiency (88 %–100 %),.^9^^,^^10^ positioning it as a valuable modality for comprehensive pelvic imaging. The non-invasive nature of MRI, combined with its ability to characterize lesion extent and venous anatomy, makes it particularly suitable for the initial evaluation of women with chronic pelvic pain.

This study has important limitations. Its retrospective design and modest sample size limit generalizability and statistical power, increasing the possibility of type II error. The endometriosis diagnosis was based exclusively on imaging findings, without histological confirmation. In addition, the absence of a control group of women with chronic pelvic pain without pelvic varicosities precludes causal inference, allowing only the identification of associations. Finally, imaging interpretation was performed by a single radiologist, which may limit interobserver reproducibility.

Future research should include prospective studies with larger sample sizes, histological confirmation of endometriosis, and appropriate control groups to better characterize the relationship between endometriosis and pelvic venous disorders. Such studies may clarify whether these conditions share common pathophysiological pathways or represent overlapping but independent contributors to chronic pelvic pain.

## Conclusion

This study demonstrates a high prevalence (61.5 %) of radiological findings consistent with endometriosis among women with confirmed pelvic varicosities. Despite inherent limitations of this retrospective analysis, the observed prevalence exceeds that reported in general symptomatic populations, warranting further investigation.

Multiparametric MRI proved effective in simultaneously identifying vascular and gynecological abnormalities, reinforcing its role as a valuable non-invasive tool for integrated assessment of chronic pelvic pain. This comprehensive imaging approach may enhance diagnostic efficiency and patient management by enabling evaluation of multiple potential pathologies in a single examination.

Although causal relationships cannot be inferred, these findings highlight the need for integrated diagnostic approaches and further investigation of potential shared mechanisms. Future prospective studies with histological confirmation and control groups are needed to better characterize the relationship between endometriosis and pelvic venous disorders and to determine optimal management strategies for women presenting with both conditions.

## Ethics approval

This study was approved by the institutional research ethics committee, in accordance with the Declaration of Helsinki and national regulations. Informed consent was waived due to the retrospective nature and use of anonymized data.

## Authors' contributions

Mira Zlotnik: Study design; data interpretation; manuscript writing, original draft.

Mira Zlotnik, Marcos de Lorenzo Messina: Data analysis; clinical correlation; manuscript revision.

Andrei Skromov de Albuquerque, Thiago Dieb Ristum Vieira: Image acquisition; radiological analysis.

Vinicius Bertoldi, Pedro Puech Leão: Vascular evaluation; critical revision of the manuscript.

Edmund Chada Baracat: Supervision; Clinical validation; final review.

All authors contributed to the final manuscript and approved its submitted version.

## Funding

No funding was received for this study.

### Data availability statement

The datasets generated and/or analyzed during the current study are available from the corresponding author upon reasonable request.

## Declaration of competing interest

The authors declare no conflicts of interest.

## Appendix Supplementary materials

Image, application 1
