# Supplementary material for: High prevalence of endometriosis in women with chronic pelvic pain and confirmed pelvic varicosities: a multiparametric MRI study
Source: Clinics (Sao Paulo). 2026 Mar 3;81:100895. doi: 10.1016/j.clinsp.2026.100895 (PMC12969381; doi:10.1016/j.clinsp.2026.100895)
Supplement: Supplementary file 1 [file mmc1.docx]

**CLINICS-D-25-01259**

**Supplementary Table**

**Table S1** Individual patient data.

|  | **Age at MRI (years)** | **Pregnancies** | **CPP (VAS)** | **Dyspareunia (VAS)** | **Dysmenorrhea (VAS)** | **Endometriosis** |
| --- | --- | --- | --- | --- | --- | --- |
| **Patient 1** | 22 | 1 | 9 | 8 | 10 | No |
| **Patient 2** | 46 | 4 | 10 | 10 | 10 | Yes |
| **Patient 3** | 42 | 4 | 8 | 8 | 10 | No |
| **Patient 4** | 43 | 2 | 6 | 7 | 8 | No |
| **Patient 5** | 19 | 0 | 10 | 10 | 9 | No |
| **Patient 6** | 40 | 2 | 10 | 10 | 10 | No |
| **Patient 7** | 46 | 4 | 0 | 9 | 0 | Yes |
| **Patient 8** | 32 | 2 | 10 | 10 | 0 | Yes |
| **Patient 9** | 26 | 2 | 8 | 10 | 10 | No |
| **Patient 10** | 30 | 3 | 10 | 10 | 10 | Yes |
| **Patient 11** | 36 | 2 | 10 | 5 | 0 | Yes |
| **Patient 12** | 43 | 3 | 10 | 7 | 10 | Yes |
| **Patient 13** | 44 | 3 | 10 | 10 | 7 | Yes |
| **Patient 14** | 38 | 3 | NI | NI | NI | No |
| **Patient 15** | 22 | 2 | 7 | 10 | 7 | No |
| **Patient 16** | 34 | 3 | 10 | 10 | 0 | Yes |
| **Patient 17** | 41 | 1 | 6 | 0 | 7 | Yes |
| **Patient 18** | 40 | NI | NI | NI | NI | Yes |
| **Patient 19** | 35 | 1 | 7 | 7 | 10 | Yes |
| **Patient 20** | 39 | 3 | 8 | 8 | 10 | Yes |
| **Patient 21** | 49 | 3 | 8 | 10 | 8 | Yes |
| **Patient 22** | 28 | 4 | 9 | 10 | 0 | Yes |
| **Patient 23** | 25 | 1 | 10 | 0 | 2 | Yes |
| **Patient 24** | 41 | 0 | 9 | 10 | 7 | Yes |
| **Patient 25** | 41 | 1 | 9 | 0 | 9 | Yes |
| **Patient 26** | 31 | 2 | 8 | 10 | 9 | Yes |
| **Patient 27** | 22 | 0 | 9 | 9 | 8 | No |
| **Patient 28** | 37 | 3 | 9 | 9 | 10 | No |
| **Patient 29** | 30 | 3 | 8 | 10 | 2 | No |
| **Patient 30** | 23 | 0 | 8 | 10 | 0 | Yes |
| **Patient 31** | 35 | 2 | 7 | 5 | 0 | No |
| **Patient 32** | 39 | 2 | 10 | 10 | 9 | Yes |
| **Patient 33** | 31 | 3 | 10 | 10 | 10 | No |
| **Patient 34** | 33 | 5 | 10 | 10 | 10 | No |
| **Patient 35** | 33 | 1 | 6 | 10 | 4 | Yes |
| **Patient 36** | 39 | 1 | 10 | 10 | 10 | Yes |
| **Patient 37** | 38 | 2 | 8 | 8 | 7 | No |
| **Patient 38** | 40 | 4 | 10 | 8 | 8 | Yes |
| **Patient 39** | 47 | NI | NI | NI | NI | No |
| **Patient 40** | 45 | 1 | 8 | 8 | 8 | Yes |
| **Patient 41** | 31 | 3 | 7 | 8 | 10 | Yes |
| **Patient 42** | 32 | 4 | 10 | 9 | 5 | No |
| **Patient 43** | 37 | 2 | 10 | 10 | 0 | Yes |
| **Patient 44** | 39 | 5 | 10 | 10 | 10 | Yes |
| **Patient 45** | 37 | 2 | 8 | 8 | 0 | Yes |
| **Patient 46** | 40 | 0 | 7 | 0 | 9 | No |
| **Patient 47** | 30 | 1 | 7 | 8 | 10 | Yes |
| **Patient 48** | 25 | 2 | 10 | 10 | 2 | Yes |
| **Patient 49** | 23 | 0 | 9 | 8 | 0 | Yes |
| **Patient 50** | 39 | 1 | 10 | 8 | 8 | No |
| **Patient 51** | 41 | 4 | 9 | 8 | 8 | No |
| **Patient 52** | 19 | 0 | 8 | 4 | 7 | Yes |

CPP, Chronic Pelvic Pain; VAS, Visual Analog Scale (0‒10); NI, Not Informed.
